# Climatic Variability Caused by Topographic Barrier Prevents the Northward Spread of Invasive *Ageratina adenophora*

**DOI:** 10.3390/plants11223108

**Published:** 2022-11-15

**Authors:** Yi Zhang, Ziyan Liao, Han Jiang, Wenqin Tu, Ning Wu, Xiaoping Qiu, Yongmei Zhang

**Affiliations:** 1 China-Croatia ‘Belt and Road’ Joint Laboratory on Biodiversity and Ecosystem Services, Chengdu Institute of Biology, Chinese Academy of Sciences, Chengdu 610041, China; 2 University of Chinese Academy of Sciences, Beijing 100049, China

**Keywords:** invasive alien species, ensemble model, spread dynamic, environmental factors, topographic barrier, ecological security

## Abstract

*Ageratina adenophora* (Spreng.) R.M.King & H.Rob. is one of the most threatening invasive alien plants in China. Since its initial invasion into Yunnan in the 1940s, it spread rapidly northward to southern Mount Nyba in Sichuan, which lies on the eastern edge of the Qinghai–Tibet Plateau. During fieldwork, we found an interesting phenomenon: *A. adenophora* failed to expand northward across Mount Nyba, even after the opening of the 10 km tunnel, which could have served as a potential corridor for its spread. In this work, to explore the key factors influencing its distribution and spread patterns, we used a combination of ensemble species distribution models with the MigClim model. We found that the temperature annual range (TAR), precipitation of driest month (PDM), highway density (HW), and wind speed (WS) were the most predominant factors affecting its distribution. The north of Mount Nyba is not suitable for *A. adenophora* survival due to higher TAR. The spatial–temporal dynamic invasion simulation using MigClim further illustrated that the northward invasion of *A. adenophora* was stopped by Mount Nyba. Overall, Mount Nyba may act as a topographic barrier that causes environmental differences between its south and north sides, preventing the northward invasion of *A. adenophora*. However, other suitable habitats on the northern side of the mountain still face challenges because *A*. *adenophora* is likely to invade via other routes. Therefore, long-term monitoring is needed to prevent human-induced long-distance spread events.

## 1. Introduction

The invasion of non-native plants has become one of the most threatening factors reducing biodiversity and altering the ecological function of invaded habitats [1,2,3,4]. *Ageratina adenophora* (Spreng.) R.M.King & H.Rob. is one of the most problematic perennial herbs. It is native to Central America and was introduced outside of its native land as an ornamental in the 19th century [5]. Due to the production and dispersal of numerous seeds, prolonged seed viability, high stress tolerance, high phenotypic plasticity, and competitive ability (such as allelopathy), *A. adenophora* has successfully invaded many countries and regions, mainly in Asia, Africa, Europe, and Oceania, and it has had serious ecological impacts and caused economic losses [6]. In China, since its first record in the southwestern province of Yunnan from Myanmar in the 1940s [7], this species spread rapidly, and over 70 million hectares has been invaded according to the census in 2015. It has been demonstrated that the soil microbial community was changed by the invasion of this alien weed, causing a radical decline in the biomass of native plants [8]. Moreover, the invasion of *A. adenophora* into grassland has indirectly led to a decline in indigenous plants and grazing animals, resulting in great economic loss in animal husbandry (RMB 0.99 million/yr) and grassland ecosystem services (RMB 2.63 million/yr) [9]. 

Previous studies on *A. adenophora* mainly focused on its biological and ecological characteristics, local impacts, and historical invasion process and the approaches to its eradication [10,11,12,13,14]. In addition, expansion mechanisms have attracted increasing attention in the past decade [15]. For instance, both environmental conditions and their biological features have been considered to influence the spread of alien plants [15]. The environment conditions include air temperature, solar radiation, precipitation, soil pH, biomass, allelopathy, inter-species competitiveness, genetic characteristics (rapid evolution and phenotypic plasticity), and human disturbance (land use and land cover change) [9,16]. 

Studies on the historical distribution of *A. adenophora* in Yunnan Province, where it originally invaded, showed that it reached an equilibrium status in the 1990s, fifty years after its invasion into this area [13]. Afterward, it rapidly invaded the neighboring provinces, such as Sichuan and Chongqing upstream of the Yangtze River, at an average expansion rate of 13.2 km·yr^−1^ [13]. However, *A. adenophora* did not occupy all the potential areas nationwide [13]. It was recorded that, in 2011, *A. adenophora* spread northeastward to Pingshan County (104.2° E, 28.6° N) and northward along the Hengduan Mountainous region to Hanyuan County (102.7° E, 29.3° N) of Sichuan Province. Hanyuan County is located on the southern side of Mount Nyba [13,15]. With a peak elevation of 3300 m above sea level, Mount Nyba is located between Hanyuan (102.65° E, 29.35° N) and Yingjing (102.85° E, 29.80° N) Counties in Sichuan. With the reported expansion rate [13], *A. adenophora* should have invaded into the northern side of Mount Nyba, and it should have even occupied the whole Chengdu Plain. However, *A. adenophora* has not been found on the northern side of Mount Nyba. Indeed, some studies using Ecological Niche Modeling (ENM) predicted that the low-altitude belts on the northern side could be potential distribution areas of the species [9,17,18]. At first, researchers thought that the high elevation of the mountain was the reason for the failed invasion of *A. adenophora* into the northern side. 

In 2012, the Traffic Tunnel of Mount Nyba was opened. Before the opening of the tunnel, a road at an altitude of 2552 m across Mount Nyba was the only traffic route linking both sides. Since the initial discovery of *A. adenophora* on the south side of Mount Nyba in 2011, there have been no records about its existence on the opposite side to date, even after the opening of the Traffic Tunnel of Mount Nyba, which could have served as a potential corridor for the plant’s spreading due to vehicles and human movement. It seems that the environmental conditions on the northern side render it unsuitable for the establishment of *A. adenophora*.

Mountain Nyba is a part of the Daxiang Mountain Range. Topographically, it is the transitional area between the Chengdu Basin and the Qinghai–Tibet Plateau, as well as the watershed between Dadu River and Qingyi River. In addition, due to the rain shadow effect of the southeast monsoon, the Daxiang Mountain Range is the climatic division between the dry area to the southwest and the humid area to the northeast of the mountain. The annual precipitation of Hanyuan County located on the south-facing slope is only 741.8 mm; Yingjing County located in the north has as much as 1133.1 mm. The great climate differences between the two sides of the mountain have resulted in differences in the vegetation types, i.e., lush forests growing on the north-facing slope and low shrubs growing on the south-facing slope (Online Resource Figure S1). 

At the average expansion rate of 13.2 km·yr^−1^, after 1990 [13], *A. adenophora* would have invaded the north side of Mount Nyba in one year after the opening of the tunnel in 2012 and would have occupied the Chengdu Plain within 20 years. However, to date, the species has not spread across the mountain. We presume that Mount Nyba causes the environmental differences between the two sides of the mountain, with these environmental differences further affecting the northward expansion of *A. adenophora*. In this work, based on the current distribution data collected from major data platforms, an ensemble model was applied to define the potential distribution of *A. adenophora* and the key influencing environmental factors. The MigClim model was used for a spread simulation of *A. adenophora*. The different environmental conditions on both sides of Mount Nyba were further analyzed to explore the possible mechanisms that prevent the northward invasion of *Ageratina adenophora* via the mountain, which is located on the eastern edge of the Qinghai–Tibet Plateau.

## 2. Results

### 2.1. Spatial–Temporal Distribution of Ageratina adenophora

**Figure 1:**
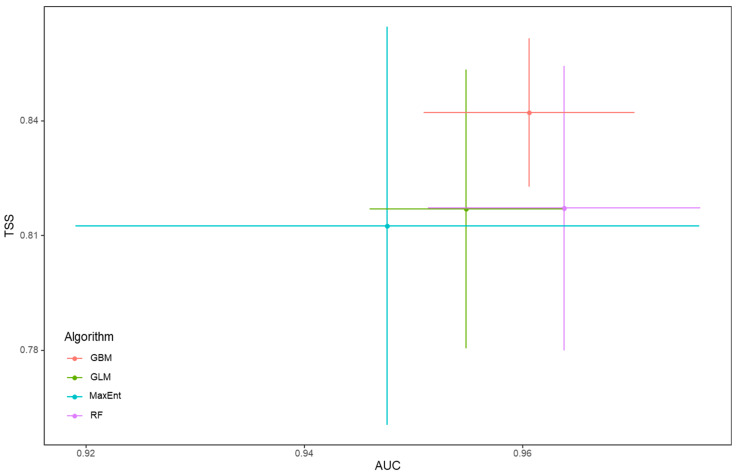
The performance of individual algorithms indicated by AUC and TSS.

**Figure 2:**
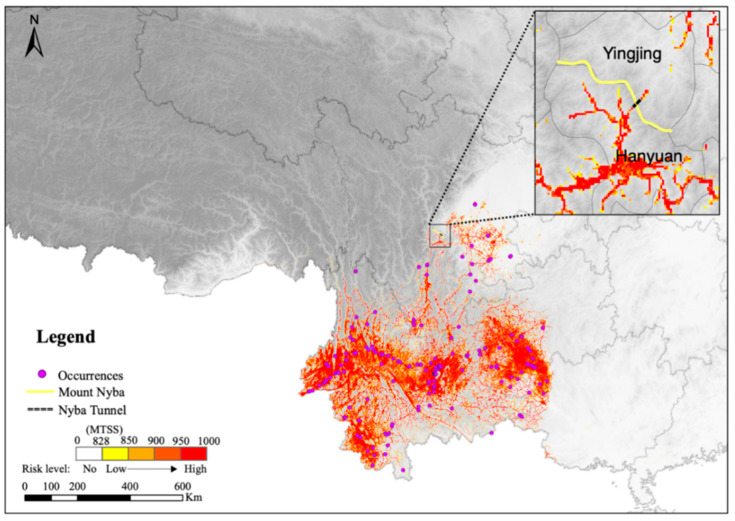
Suitable habitats of *A. adenophora* predicted using ensemble model within study area.

The mean AUC values of the generalized linear model (GLM), generalized boosting model (GBM), random forest (RF), and maximum entropy (MaxEnt) were 0.95, 0.96, 0.96, and 0.95, respectively, which were all greater than 0.90 (Figure 1). Thus, all four of the species distribution models were usable in the final ensemble model for assessing the habitat suitability of *A. adenophora*. The mean AUC and TSS values of the final ensemble model were 0.96 and 0.82, respectively, indicating a satisfactory accuracy of the predicted results. The total potential suitable distribution region of *A. adenophora* in the study area (suitability > MTSS) reached 3.31 × 10^5^ km^2^, mainly in the western and eastern parts of Yunnan, as well as in the southwestern parts of both Yunnan and Guizhou (Figure 2). The high-risk region occupied 53.40% (1.77 × 10^5^ km^2^) of the entire suitable habitat of *A. adenophora*, and the moderate-risk and low-risk regions accounted for 34.22% (1.13 × 10^5^ km^2^) and 12.40% (4.10 × 10^4^ km^2^), respectively. It was noted that the north side of Mount Nyba is not suitable for the growth of *A. adenophora* according to the prediction.

### 2.2. Determination of the Key Environmental Factors

**Figure 3:**
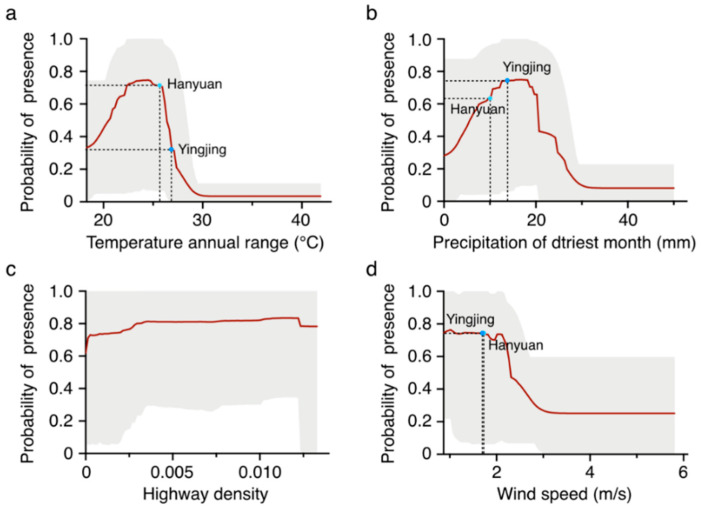
Response curves of key variables influencing *A. adenophora* distribution. The average values of all four variables in Hanyuan and Yingjing are marked. (**a**) Response curve of temperature annual range (°C). (**b**) Response curve of precipitation of driest month (mm). (**c**) Response curve of highway density. (**d**) Response curve of precipitation of wind speed (m/s).

Among the 16 analyzed variables, the temperature annual range (TAR), precipitation of driest month (PDM), highway density (HW), and wind speed (WS) were found to be the top 4 important predictors in the ensemble model, despite the large variation in predictor importance between algorithms (Table 1). The temperature annual range (TAR) was the most predominant environmental factor affecting the existence probability of *A. adenophora* in all the models used, except for RF (Table 1). In the final ensemble model, the permutation importance of TAR was 0.47, followed by PDM (0.24), HW (0.13), and WS (0.13) (Table 1). According to the response curves of the key variables, the thresholds (existence probability > MTSS) were 20.95–26.30 °C for TAR, 6.00–20.45 mm for PDM, 0–1.00 for HW, and 0–2.29 m/s for WS (Figure 3). In addition, the presence probability of *A. adenophora* showed a similar change trend responding to TAR and PDM, which increased gradually at first and plummeted after meeting the upper threshold (Figure 3a,b).

### 2.3. Comparison of Environmental Variables between Northern and Southern Sides of Mount Nyba

Based on the prediction by our ensemble model, the north of Mount Nyba is not suitable for *A. adenophora* survival (Figure 2), which likely results from the different environmental conditions between the two sides of the mountain. For an environmental comparison, Hanyuan and Yingjing Counties were chosen as representative regions on the south and north sides of Mount Nyba, respectively. Among the four key factors, TAR, PDM, and WS were significantly different between Hanyuan and Yingjing (Online Resource Figure S3). Combined with the response curves, we found that the unsuitability of Yingjing for *A. adenophora* survival was mostly affected by the most important variable, TAR. The TAR values in Yingjing ranged from 25.30 °C to 27.80 °C, and 86.72% of the area is beyond the thresholds suitable for *A. adenophora* survival (20.95–26.30 °C). In comparison, the TAR values in Hanyuan ranged from 24.90 °C to 26.90 °C, and 92.03% of Hanyuan is within the suitable thresholds. The mean values of TAR in Yingjing and Hanyuan were 26.86 °C and 25.81 °C, respectively (Figure 3 and Online Resource Figure S3). The corresponding presence percentage of *A. adenophora* in Yingjing was around 0.30, while it was above 0.70 in Hanyuan (Figure 3a). Some other environmental factors were also different between Yingjing and Hanyuan, but they were less influential on the potential distribution of *A. adenophora* (Figure 3 and Online Resource Figure S3). Taken together, the north side of Mount Nyba is not suitable for *A. adenophora* growth, and this is mainly caused by the north side having a higher TAR than the south side. 

### 2.4. Reconstruction of A. adenophora Expansion History in Study Area

**Figure 4:**
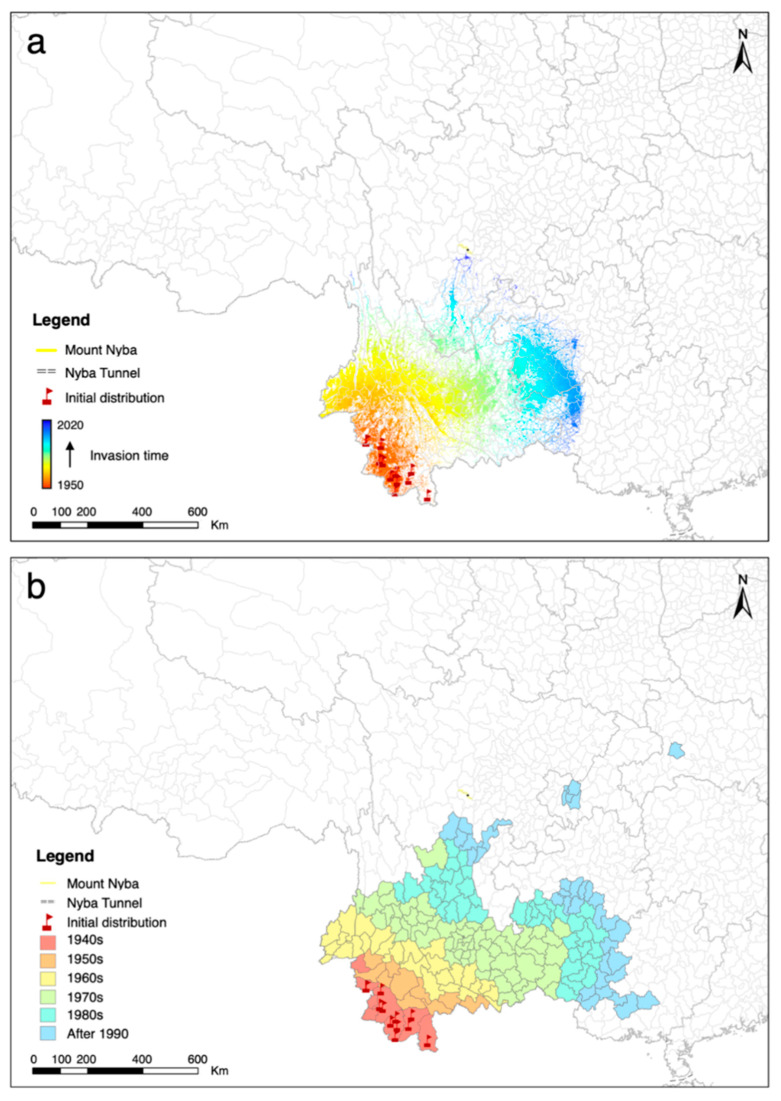
Comparison between estimated and investigated expansion of *A. adenophora*. (**a**) The spatially explicit expansion process of *A. adenophora* over 70 years (1950–2020) in study area simulated using MigClim model. (**b**) Expansion of *A. adenophora* roughly investigated in a previous study [13].

To further clarify the expansion history of *A. adenophora*, MigClim was used to simulate its spread for 70 y based on the initial occurrence, habitat suitability from the ensemble model, and both short- and long-distance dispersal scenarios (Table 2 and Online Resource Table S1). The dynamic expansion process of *A. adenophora* in each year from 1950 to 2020 with a spatial resolution of 1 km was obtained (Figure 4a and Online Resource Video S1). The estimated expansion process was consistent with the investigative spread in a previous study (Figure 4b) [13], indicating the accuracy of the model and parameters used in this study. The results show that, after its initial invasion in the south of Yunnan, *A. adenophora* spread quickly to the north and east and occupied the suitable habitats (Figure 4a and Online Resource Video S1). It was observed that *A. adenophora* invaded north from Yunnan to Sichuan and reached the south of Mount Nyba. Then, it stopped its northward expansion across the mountain and turned to the east (Online Resource Video S1), which is consistent with the unsuitability of the north side for *A. adenophora* survival (Figure 2). In addition to the northward expansion, *A. adenophora* also invaded the east of Guizhou and Guangxi (Figure 4a and Online Resource Video S1).

## 3. Discussion

### 3.1. Distribution and Spread Prediction of A. adenophora

Projecting the potential habitats of invasive alien plants is an important basis for formulating appropriate monitoring and management practices. A variety of methods have been developed for habitat distribution modeling. The predictive power of modeling varies due to the use of different algorithms and data processing methods [23]. The ensemble modeling approach combined with several algorithms is an increasingly used method, which can avoid uncertainty caused by model selection and obtain more reliable predictions [24]. In this study, GLM, GBM, RF, and MaxEnt were combined, and the AUC and TSS statistics results (0.96 and 0.82, respectively) showed that the final ensemble model had good discriminability for predicting *A. adenophora* distribution. It was predicted that *A. adenophora* was likely to distribute widely in southwest China, and the total area (suitability > MTSS) reached 3.31 × 10^5^ km^2^. Southwest China, including Sichuan and Yunnan, has plenty of unique landforms, such as the Sichuan Basin, the Hengduan Mountains, and the Tibetan Plateau [25,26], which provide not only diverse habitats for rich biodiversity but also suitable space for the growth of alien species.

As a very invasive species, *A. adenophora* spread into southwest China from south to north in the last 60 years and finally occupied a vast ‘disaster area’ [13,17]. In this analysis, it is important to clarify the expansion processes of invasive alien plants for further management [27]. The expansion of *A. adenophora* is not only affected by environmental features but also by its own reproductive and invasive characteristics. The MigClim R package was designed to combine species-specific dispersal information, including propagule production ability, dispersal distance and kernel, long-distance dispersal, and barriers to dispersal, with habitat invasibility to obtain distribution and spread patterns with high reliability [19]. The expansion process reconstructed in this work is consistent with the investigations in the previous study [13], indicating the optimum parameters used in MigClim and the accuracy of our model in *A. adenophora* expansion estimation. In addition, the spatial resolution of the input data in MigClim was 1 km, and the expansion map of *A. adenophora* can be updated every year. With high accuracy, we can clearly illustrate the historical expansion process of *A. adenophora* for each year, which is helpful to explore the complex expansion mechanisms. Once an understanding of the potential distribution is achieved, the next step is the estimation of future expansion under environmental change conditions, which can also be modeled by MigClim with the parameters used in this study. Based on those rational predictions, highly effective monitoring and appropriate management measures can be expected.

### 3.2. Key Driving Factors

As one of the most crucial factors, climate plays a key role in the geographic distribution of plant species and vegetation patterns [28,29]. It is crucial to clarify the key driving environmental factors influencing the patterns and spread of the invading species for managing it effectively [30]. Among the 16 analyzed variables, TAR, PDM, HW, and WS were the predominant ones in controlling *A. adenophora* potential spreading. In particular, TAR and PDM were the top two important factors in three of the four selected models, and *A. adenophora* distribution probability showed a sharp decline with an increase in TAR and PDM, indicating that *A. adenophora* is quite sensitive to changes in temperature and precipitation. However, it has been reported that *A*. *adenophora* prefers regions with greater precipitation [31], which does not quite agree with our results. In fact, in a certain range (PDM < 20.45 mm), the suitability for *A. adenophora* did increase with an increase in precipitation, but when it continued increasing, the region became unfit for *A. adenophora* growth (Figure 3).

Highway density was found to significantly influence *A. adenophora* distribution in this study. Southwest China is a tourist hub greatly affected by human activities. The frequency of the intentional or unintentional introduction of alien plants in this region has been found to be relatively high [32]. Dense traffic roads might cause habitat fragmentation and disturbance, and the resultant empty niche creates opportunities for the invasion of alien plants [33]. In addition, frequent transportation increases the chance of the spreading of plant seeds or other types of propagules [34]. Although highway density was correlated with the distribution of *A*. *adenophora*, it did not affect the suitability as much as other key factors.

The direct effects of topographic variables (slope and aspect) were not observed to influence the distribution of *A. adenophora* in our study. The lack of direct effect of topographic variables may reflect the limited spatial scale of the predictors (the resolution of the raster data was 1 km) used in the analysis.

### 3.3. Possible Prevention Mechanism of A. adenophora Invasion by Mount Nyba

Studies on the origin and invading locations of invasive plants have indicated that invasive alien plants first colonize an environment similar to their original environment, but this is not always true for their subsequent expansion because invasive alien plants have a large ecological niche and high adaptability to newly invaded places [35]. The origin of *A. adenophora* is Central America, in which the climatic conditions are similar to those in Yunnan. Starting from Yunnan to Mount Nyba, the climate change alongside the northward invading route of *A. adenophora* is mild, which guarantees the colonization and growth of the plant. However, the invasion of *A. adenophora* stopped when it reached the south of Mount Nyba. Based on our predictions using the ensemble model, the north side of Mount Nyba is not suitable for *A. adenophora* growth. Mount Nyba resists the rain shadow effect of the southeast monsoon, which causes different climates and vegetation between the two sides of the mountain. The temperature annual range (TAR) is one of the most important environmental variables. As mentioned above, the TAR value of 86.72% of Yingjing (north side of Mount Nyba) is above the upper threshold (26.30 °C), being too high for *A. adenophora* to survive. While in Hanyuan (the south side of Mount Nyba), the TAR value of over 90% in the area is within the suitable thresholds. In addition to the high TAR, there may be many other factors contributing to the failed invasion of *A. adenophora* to the north side.

The production of numerous fruits is one of the biological characteristics of *A. adenophora* conducive to successful invasion [6]. The light fruits can be easily dispersed along rivers and roads, which facilitates its invasion of new habitats [15]. In addition, the same germination vigor has been observed in the seeds from central and marginal florets [36]. Moreover, ungerminated seeds provide the main source for the formation of a stable seed soil bank, serving as a potential population of *A. adenophora* [37]. However, it is worth noting that the opening of the Traffic Tunnel of Mount Nyba in 2012 did not accelerate *A. adenophora* spread to the northern side of the mountain. It has been reported that the germination of *A. adenophora* seeds could be affected by several environmental factors [37,38,39]. Lower light has been found to markedly restrict *A. adenophora* seed germination [38,39]. According to our data, the solar radiation in Yingjing is much lower than that in Hanyuan (Online Resource Figure S4). Although it is possible for *A. adenophora* fruits to be dispersed by animals, wind, and vehicles through the Traffic Tunnel of Mount Nyba, the lower solar radiation in Yingjing might negatively affect the seed germination rates of *A. adenophora*. In addition, the north-facing slope is moister and grows lush forests, while the south-facing slope is drier and grows low shrubs (Online Resource Figure S1). Forests with luxurious branches and leaves could inhibit the growth of *A. adenophora* mainly due to the weak light intensity [40]. Taken together, the different environmental conditions might be responsible for the failure of *A. adenophora* invasion on the northern side of Mount Nyba by inhibiting the establishment stage.

### 3.4. Prevention and Control Methods

The Chengdu Plain is one of the major agricultural areas in the southwest of China, and it is also suitable for *A*. *adenophora* growth. Once invaded by *A*. *adenophora*, great losses will be caused. The presence of Mount Nyba does prevent the northward invasion of *A*. *adenophora* to the Chengdu Plain; however, the area still faces challenges because *A*. *adenophora* is likely to invade this area through other routes. For example, it can spread from northeastern Yunnan or northwestern Guizhou to the Chengdu Plain (Figure 4 and Online Resource Video S1). Furthermore, over long-term evolution and development, every plant species interacts with external environmental factors and establishes numerous strategies in order to enhance its ability to evolve in response to environmental heterogeneity [41]. Recent studies have reported that some invasive plants spread into new areas by rapidly changing their genes [42]. It is possible for *A*. *adenophora* to produce seeds with higher germination activity and survivability. With the adaptive evolution of *A*. *adenophora*, if the colonization is successfully established on the north side of Mount Nyba, *A. adenophora* will continue moving northward at a very fast speed, which might be a great threat to the Chengdu Plain area. Once an invasive plant establishes a viable population in a new habitat, it is difficult to eradicate it [43]. The management is not only expensive but also very limited because it can only attenuate further harm rather than restore the ecological environment to its original state [44,45]. In comparison, prevention or preclusion is a cheaper, eco-friendly, and more effective way to minimize the damage caused by invasive plants [33,46]. Based on the findings and conjecture of this work, the following measures can be taken to preclude the expansion of *A*. *adenophora*: (1) investigate the detailed distribution and potential spread routes of *A*. *adenophora* in southwest China, and put forward specific control strategies based on the bioecological characteristics and adaptation to the local environment; (2) keep monitoring those regions on the estimated spread routes with a high invasion risk for the early detection of new invasion and to take remedial action in a timely manner; (3) reduce the level of human disturbance on susceptible ecosystems forecasted by this study, and alter land use accordingly, such as by returning farmland to forests, to maintain ecological diversity; and (4) carry out a risk assessment of *A*. *adenophora* during road construction and urban planning. The measures mentioned above also can be widely applied to avoid the invasion of other alien plants in regions with potential risk.

## 4. Materials and Methods

### 4.1. Occurrence Data of Ageratina adenophora

The occurrence data of *A. adenophora* were collected from the following websites: Global Biodiversity Information Facility (GBIF, Copenhagen, Denmark; https://doi.org/10.15468/dl.pt3bhu (accessed on 22 June 2022)), National Specimen Information Infrastructure (NSII, http://www.nsii.org.cn (accessed on 22 June 2022)), and the National Plant Specimen Resource Centre (NPSRC, http://www.cvh.ac.cn (accessed on 22 June 2022)). Additional occurrence records were extracted from related published studies. To minimize the effect of spatial autocorrelation on the modeling results, ArcGIS 10.3 (ESRI, Redlands, CA, USA) was used to confirm that each grid cell (1 km × 1 km) had only one occurrence. A total of 171 occurrences of *A. adenophora* were used for ensemble modeling (Online Resource Figure S2 and Online Resource Table S1).

### 4.2. Environmental Data

Thirty initial eco-geographic data were selected in the modeling process to preliminarily evaluate ecological niche dimensions. Among them, 19 bioclimatic variables [47], 2 topographic variables (slope and aspect index), solar radiation (SR), and wind speed (WS) were downloaded from Worldclim (https://www.worldclim.org (accessed on 23 June 2022)); soil type was identified by the Third Pole Environment Data Center (TPEDC, http://westdc.westgis.ac.cn/zh-hans (accessed on 23 June 2022)); land cover classification was based on the satellite imagery of Advanced Very High Resolution Radiometer (AVHRR) for 2015 from Finer Resolution Observation and Monitoring of Global Land Cover (FROM-GLC, http://data.ess.tsinghua.edu.cn (accessed on 23 June 2022)); two anthropogenic factor data (population density and human interference index) were obtained from Socioeconomic Data and Applications Center (SEDAC, https://sedac.ciesin.columbia.edu (accessed on 23 June 2022)); and two traffic data (main highway density and main railway density) and river data were taken from National Catalogue Service for Geographic Information (NCSGI, https://www.webmap.cn (accessed on 23 June 2022)). The range of the study area was limited to 85.73–113.01° E and 19.59–36.49° N (Online Resource Figure S2), and the resolution of all grids was 1 km × 1 km. Some studies indicated that strong collinearity between pairwise variables might lead to the misinterpretation of modeling results [48,49,50]. To ensure that each environmental predictor was independent from others, we checked the variance inflation factor (VIF) values by taking all 30 variables into the *vifstep* function of usdm R package [51]. Any variable with a VIF value above 5 was excluded from the predictor sets based on backward stepwise regression [50]. Finally, a total of 16 variables, namely, precipitation seasonality (PS), isothermality (ISO), precipitation of driest month (PDM), precipitation of warmest quarter (PWQ), temperature annual range (TAR), solar radiation (SR), highway density (HW), railroad density (RAD), soil type (ST), land use/land cover (LULC), wind speed (WS), slope (SLO), aspect index (AI), river density (RD), human interference index (HII), and population density (PD), were used for modeling (Table 1). 

### 4.3. Habitat Suitability Simulation 

We used an ensemble approach to assess the habitat suitability for *A. adenophora*, and this approach averaged predictions across four commonly used species distribution models [52,53], namely, the generalized linear model (GLM), generalized boosting model (GBM), random forest (RF), and maximum entropy (MaxEnt). The occurrence data of the species under investigation and the environmental variables were imported into each algorithm mentioned above for the prediction, whereas every occurrence data were accompanied with a whole set of environmental variables, and the latter were, in turn, utilized to predict the potential distribution of the species. At the same time, the applicability value of each grid cell for the growth of the species was evaluated using the environmental variables within that cell. The applicability value ranged from 0 to 1000, representing the suitable degree from a totally unsuitable habitat to a totally optimal habitat. We performed this ensemble modeling in biomod2 R package [54] with some species-specific settings: (1) 10,000 pseudo-absences were randomly selected for each model replication; (2) the prevalence was weighted to 0.5 between presences and pseudo-absences; (3) a 5-fold cross-validation method was used, which randomly selected 80% of the occurrences for training and the remaining 20% for testing; and (4) the committee averaging method was used to ensemble the output of each model. Models with an AUC value less than 0.8 were excluded from the final ensemble model. According to the maximum training sensitivity plus specificity logistic threshold (MTSS, 828), these pixels were grouped into four classes: no risk (<828), minimal risk (828–850), moderate risk (850–950), and high risk (>950). A threshold-independent Receiver-Operating Characteristic Analysis (ROC) was carried out, and true skill statistics (TSSs) were calculated to calibrate the ensemble model and to validate its robustness [55]. 

### 4.4. Identification of Key Environmental Factors Influencing A. adenophora Distribution

To identify the key factors affecting the distribution of invasive alien plants, the permutation importance in all the algorithms was analyzed. Permutation importance was analyzed using Jackknife to rank the relative importance of the variables [56].

The purpose of the Jackknife procedure is to reduce the bias in the estimation, by which each variable is used alone to construct one set of models firstly, and then another set of models is constructed using all the variables together with a particular selected variable varying (in this case, all the other variables are of their average sample value) [57,58]. We used the different response curves obtained from the Jackknife procedure to show the change in the predicted existence probability with environmental variables and to analyze the relationship between the predicted existence probability and the key environmental factors.

### 4.5. Spread Simulations

To understand the spatial–temporal dynamic process of *A. adenophora* invasion, we used the MigClim model [19] to run a spread simulation based on the predicted habitat suitability and the initial distribution in the 1940s (shown in Online Resource Table S1, referring to the initial invasion time of *A. adenophora* in Southwest China) [13]. Specifically, to successfully simulate the spread process, we collected some key population parameters of *A. adenophora*, including dispersal distance and kernel, long-distance dispersal, propagule production potential, and habitat invasibility (Table 2).

## 5. Conclusions

This study, for the first time, quantitatively depicts the year-by-year invasion dynamics of a globally hazardous invasive plant, *A. adenophora*, in China based on both modeling approaches and empirical evidence. To examine the reasons for the differential distribution of *A. adenophora* on the north and south sides of Mount Nyba, the ensemble modeling approach was chosen to identify the potential regions vulnerable to *A. adenophora* invasion and the key influencing factors. Based on the results, the temperature annual range (TAR), precipitation of driest month (PDM), highway density (HW), and wind speed (WS) were the most dominant factors. As affected by topographically determined climate patterns, the northern side of Mount Nyba showed a higher temperature annual range than the southern side, which significantly reduced the survival property and slowed the northward expansion of *A. adenophora*. In addition, we reconstructed the expansion process of *A. adenophora* with high precision, clarifying the spread of *A. adenophora* in each year from 1950 to 2020 with a spatial resolution of 1 km. Our results provide a further understanding of the expansion mechanisms of *A. adenophora*, and they may help in estimating its potential spread route and in making plans for effective monitoring along with appropriate prevention and control measures. Our results provide clues for controlling *A. adenophora* in the Chengdu plain: (1) The north of Mount Nyba is important for the monitoring of the plant, which should be cleared once spotted. (2) Human disturbance should be reduced in this area while altering land usage accordingly, such as returning farmland to forests in order to maintain ecological diversity.

## Figures and Tables

**Table 1:** Permutation importance of the variables used in the modeling for *A. adenophora* (resolution: 1 km × 1 km).

Code	Name	Unit	Importance					VIF
			GLM	GBM	RF	MaxEnt	Ensemble	
ISO	Isothermality	(*100) -	0.04	0.01	0.20	0.07	0.08	3.1
PDM	Precipitation of driest month	mm	0.41	0.19	0.04	0.31	0.24	4.1
HW	Highway density	1	0.04	0.07	0.18	0.25	0.13	1.1
TAR	Temperature annual range (bio05-bio06)	°C	0.54	0.65	0.17	0.53	0.47	5.0
LULC	The land use/land cover	-	0.01	0.00	0.05	0.09	0.04	1.5
PS	Precipitation seasonality (coefficient of variation)	1	0.13	0.00	0.05	0.10	0.07	3.8
ST	Soil type	-	0.01	0.00	0.01	0.04	0.02	1.6
SR	Solar radiation	kJ m^−2^ day^−1^	0.02	0.00	0.04	0.03	0.02	2.5
PD	Population_density	1	0.02	0.05	0.09	0.31	0.12	1.3
WS	Wind speed	m/s	0.27	0.12	0.02	0.13	0.13	4.1
PWQ	Precipitation of warmest quarter	mm	0.02	0.01	0.03	0.13	0.05	4.6
RD	River_density	1	0.01	0.00	0.00	0.02	0.01	1.0
AI	Aspect_index	1	0.00	0.00	0.01	0.01	0.01	1.0
SLO	Slope	°	0.01	0.02	0.01	0.05	0.02	1.3
HII	Human interference index	1	0.01	0.00	0.10	0.28	0.10	2.1
RAD	Railway density	1	0.00	0.00	0.00	0.01	0.00	1.0

**Table 2:** Parameters used in the MigClim model for *A. adenophora.*

Parameter	Value or Setting	Meaning	Ref.
rcThreshold	828	MaxTSS	Simulated in this study
envChgSteps	1	Without environmental change	[19]
dispSteps	70	Update times of the habitat suitability layer	From 1940s to current, ca. 70 years
dispKernel	c(1,0.607,0.368,0.223,0.135,0.082,0.05,0.03,0.018,0.011,0.007,0.004,0.002,0.002)	Probability of a source cell to disperse *A. adenophora* as a function of distance	14 km/yr for short-distance events [13]
iniMatAge	1	Newly colonized cells are ready to produce propagules after one year	[15]
propaguleProd	c(0.02,0.25, 0.90,0.99)	Probability of an occupied cell to produce *A. adenophora* as a function of time	[20,21,22]
lddFreq	0.96	High occurrence probability of long-distance dispersal events	[15]
lddMinDist	15	The minimum distance of long-distance dispersal events	[13,15]
lddMaxDist	88	The max distance of long-distance dispersal events	[15]
replicateNb	10	The spread simulation is replicated 10 times	[19]

## Data Availability

The data used for modeling were deposited in Dryad (https://doi.org/10.5061/dryad.sj3tx9668).

## Supplementary Materials

The following supporting information can be downloaded at: https://www.mdpi.com/article/10.3390/plants11223108/s1, Table S1: The occurrence data of *A. Adenophora*; Figure S1: Vegetation types on the south- and north-facing slopes of Mount Nyba. (a) Low shrubs growing on the south-facing slope. (b) Lush forests growing on the north-facing slope. Both pictures were captured on January 26, 2022; Figure S2: Geographical locations of records of *A. adenophora*. The study area was limited to 85.73–113.01° E and 19.59–36.49° N, marked by red frame; Figure S3: Comparison of key variables between Hanyuan and Yingjing Counties. Student’s *t*-test: ** *p* < 0.01; Figure S4: Comparison of solar radiation between Hanyuan and Yingjing Counties. Student’s *t*-test: ** *p* < 0.01; Video S1: Dynamic expansion process of *A. adenophora* in each year from 1950 to 2020 using the MigClim model.
