# Supplementary material for: Climatic Variability Caused by Topographic Barrier Prevents the Northward Spread of Invasive Ageratina adenophora
Source: Plants (Basel). 2022 Nov 15;11(22):3108. doi: 10.3390/plants11223108 (PMC9695367; doi:10.3390/plants11223108)
Supplement: Supplementary file 1 [file plants-11-03108-s001.zip › Supplementary figures.pdf]

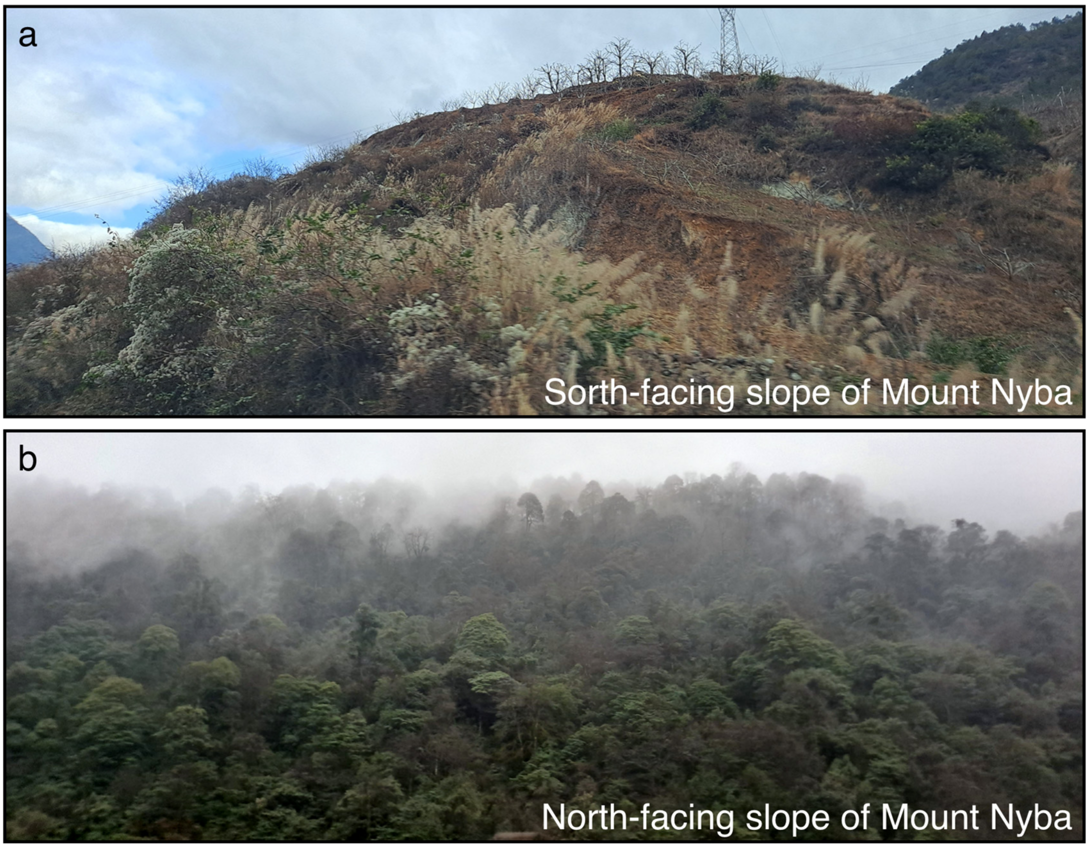

Figure S1 Vegetation types of the south- and north-facing slope of Mount Nyba. (A) Low shrubs growing on the south-facing slope. (B) Lush forests growing on the north-facing slope. Both pictures were captured on January 26, 2022

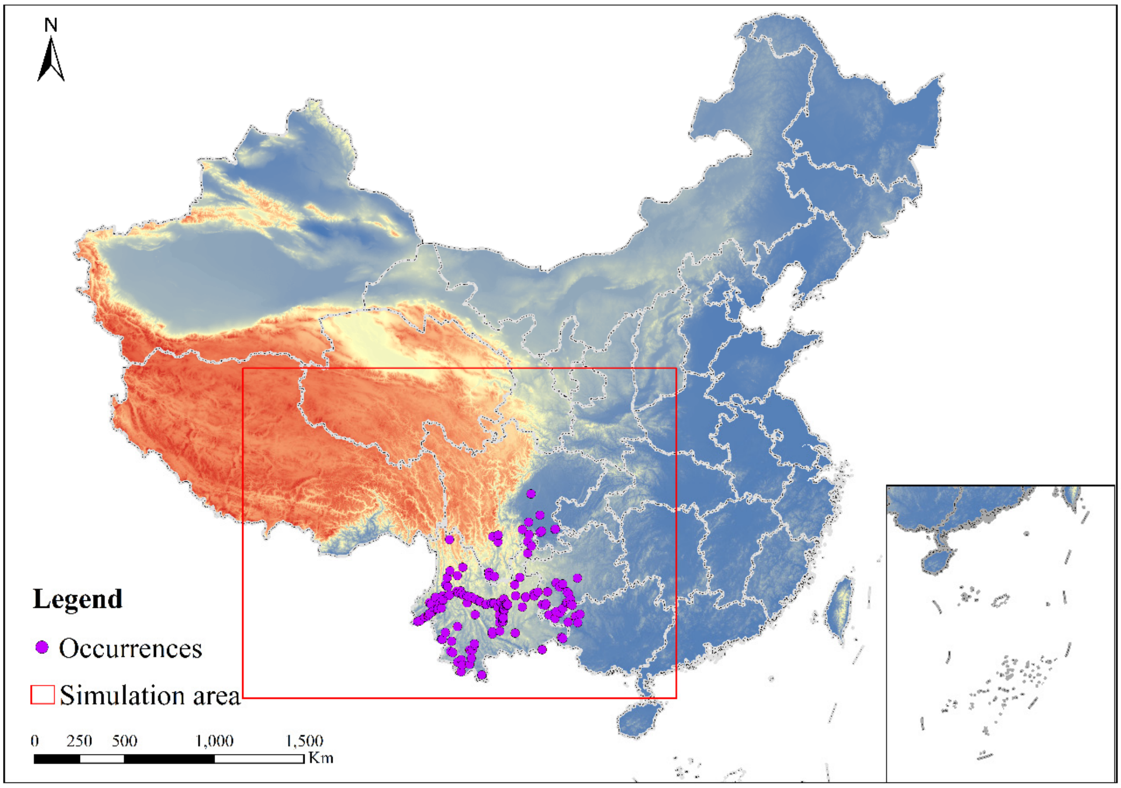

Figure S2 Geographical locations of records of *A. adenophora*. The study area was limited to 85.73 - 113.01°E and 19.59 - 36.49°N, marked by red frame

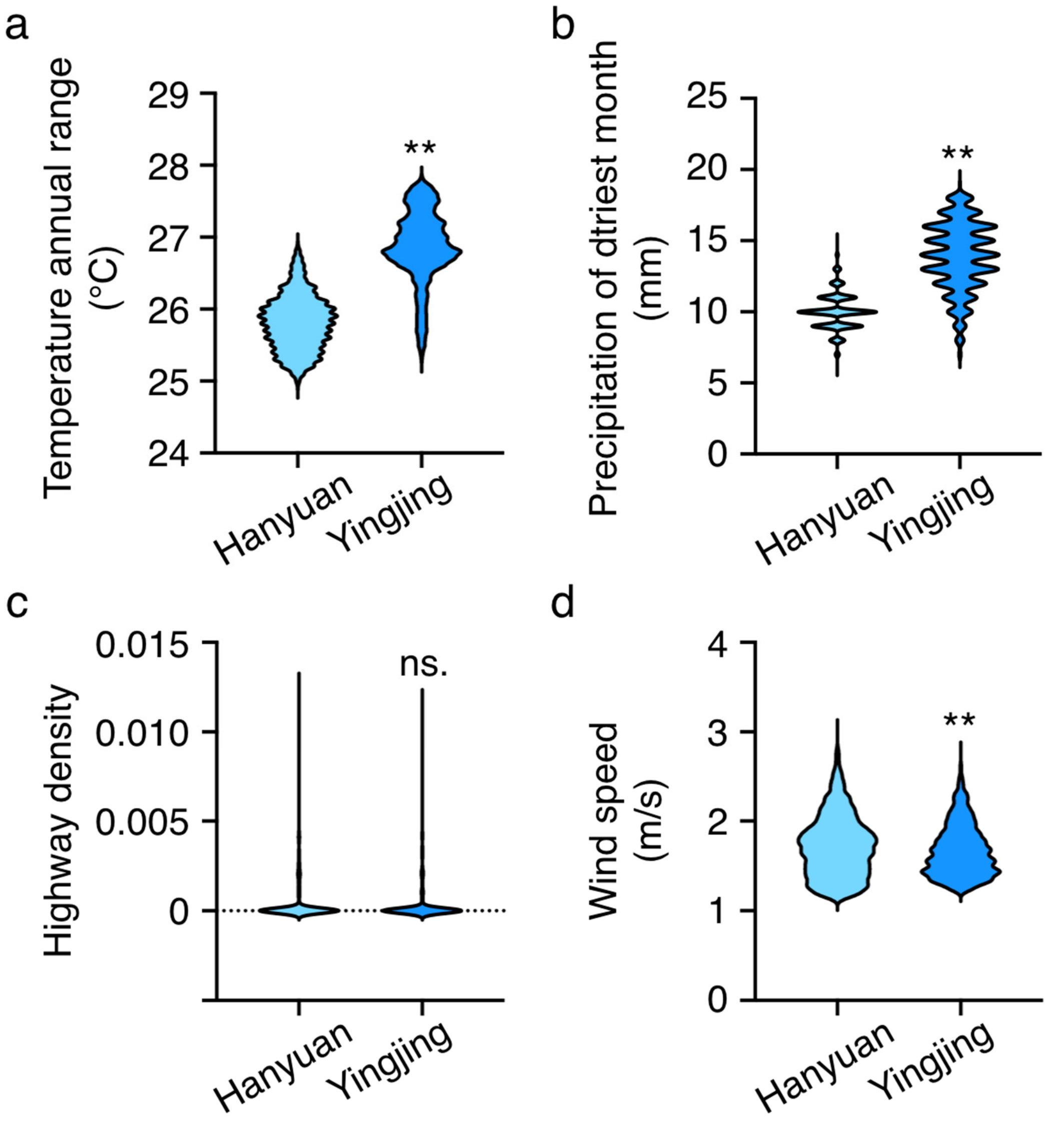

Figure S3 Comparison of key variables between Hanyuan and Yingjing County. Student's *t*-test: $**P < 0.01$

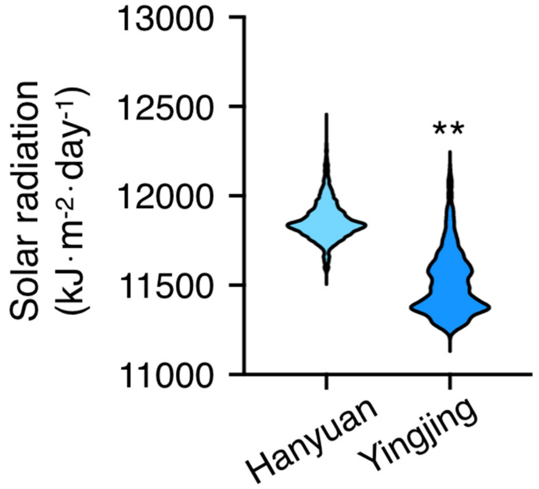

Figure S4 Comparison of solar radiation between Hanyuan and Yingjing County. Student's *t*-test:

$**P < 0.01$
